# Clinical data mining: challenges, opportunities, and recommendations for translational applications

**DOI:** 10.1186/s12967-024-05005-0

**Published:** 2024-02-20

**Authors:** Huimin Qiao, Yijing Chen, Changshun Qian, You Guo

**Affiliations:** 1 Medical Big Data and Bioinformatics Research Centre, First Affiliated Hospital of Gannan Medical University, Ganzhou, China; 2 School of Public Health and Health Management, Gannan Medical University, Ganzhou, China; 3 School of Information Engineering, Jiangxi University of Science and Technology, Ganzhou, China; 4 Ganzhou Key Laboratory of Medical Big Data, Ganzhou, China

**Keywords:** Clinical data mining, Transformative application, Heterogeneity, Analytic workflow, Predictive model

## Abstract

Clinical data mining of predictive models offers significant advantages for re-evaluating and leveraging large amounts of complex clinical real-world data and experimental comparison data for tasks such as risk stratification, diagnosis, classification, and survival prediction. However, its translational application is still limited. One challenge is that the proposed clinical requirements and data mining are not synchronized. Additionally, the exotic predictions of data mining are difficult to apply directly in local medical institutions. Hence, it is necessary to incisively review the translational application of clinical data mining, providing an analytical workflow for developing and validating prediction models to ensure the scientific validity of analytic workflows in response to clinical questions. This review systematically revisits the purpose, process, and principles of clinical data mining and discusses the key causes contributing to the detachment from practice and the misuse of model verification in developing predictive models for research. Based on this, we propose a niche-targeting framework of four principles: Clinical Contextual, Subgroup-Oriented, Confounder- and False Positive-Controlled (CSCF), to provide guidance for clinical data mining prior to the model's development in clinical settings. Eventually, it is hoped that this review can help guide future research and develop personalized predictive models to achieve the goal of discovering subgroups with varied remedial benefits or risks and ensuring that precision medicine can deliver its full potential.

## Background

Big Data is currently reinventing medicine. Clinical management has undergone a digital transformation, leading to a vast array of data known as real-world data, ranging from electronic health records (EHR) of disease phenotypes [1, 2] to the molecular atlas of patient-generated information [3], despite the acknowledged restrictions in comparison with randomized controlled trials (RCTs). Surveillance, Epidemiology, and End Results [4] is the most noteworthy example of the EHR data, while The Cancer Genome Atlas [5] represents the latter. Even the data obtained from comparative studies in which randomization is used also becomes an increasingly important source of clinical data mining [6]. With deeper involvement of machine learning, the availability of these data has led to the rapid adoption of data mining in medicine, demonstrating the prospects of developing predictive models [7–9], assessing patient risks [10–12], and facilitating physicians’ clinical decisions [13, 14]. For example, it has become possible to predict the cytotoxicity of silver nanoparticles, which are biosynthesized with anti-cancer and antibacterial activity [15–17]. Through a systematic review and statistical integration of silver nanoparticle cytotoxicity data, machine learning model training and development on these aggregated data pools can enhance the precision of risk prediction and avoid over- or underestimation of the actual risk of human exposure to nanotoxicity [18]. Although there is potential to guide precision therapies, improve efficiency, and achieve better outcomes, limited progress has been made to deal with decision-making in the clinical context.

In clinical data mining research, two perennial concerns of clinicians experienced in clinical practice have not been addressed thoroughly. The first is that data mining takes place only when the data is available rather than when the clinical needs arise, due to absence of the clinician’s active cooperation [19, 20]_._ Still, the invigorating works of data mining with active involvement of the experienced physician have been accepted by clinical guidelines [21, 22], suggesting that data mining is merging with medical practice in a fascinating way of multidisciplinary integration and raising situations in which clinical actual needs may not yet be the leading strength but increasingly become an important part of clinical data mining. The second concern is that the exotic predictive model of data mining, which has been internally or externally validated by the research conductor, does not work for current patients in the local hospital [23–26].

Recently, well-established standards for clinical data mining such as STROBE [27], TRIPOD [28] and regulatory requirements for prediction model approval from the Food and Drug Administration [29] have been available to rely on. Yet, with the dispute over "the rigor of regulations such as ENCePP [30], scholars have also questioned their feasibility [31]_._ As a result, it is easy to form the perception that external validation with favorable performance for prediction models does not prove universal applicability, considering the heterogeneity in spatial, temporal, and healthcare contexts.

Given these concerns and the purpose of our review, we conducted a systematic literature search on PubMed for articles published from 1997 to 2023, using the Medical Subject Headings (MeSH) terms "Data mining" or "Prediction model". After reviewing, we propose a frame of four principles: Clinical Contextual, Subgroup-Oriented, Confounder- and False Positive-Controlled (CSCF), to provide guidance for clinical data mining prior to the model's implementation in clinical settings. The CSCF principles are as understandable as possible by individuals engaged in data mining and are held to traditional clinical research standards. Our aim is not to substitute these established authoritative regulations with another batch of such guidelines. Rather, the target is to recognize the leading principles of clinical data mining and propose conceptual innovations that robust analytic workflows fixing a clinical problem should be serviceable and transplantable more than developed models that can be used by clinicians. Although not exhaustive, the CSCF principles can not only maximize the authenticity of developing model workflows and their products in clinical data mining, but also endow them with improved clinical outcomes when implemented in practice.

## Represent accurately and integrate into clinical practice seamlessly

There is no exception in clinical medicine where a large volume of data is generated from Hospital Information Systems, including but not limited to the Electronic Health Records (EHRs), Laboratory Information Systems, and Picture Archiving & Communication Systems. With such a large volume of data mining, many clinical questions could be addressed by developing predictive models. The use of predictive models in clinical settings includes, but is not limited to, curable factors [32], diagnosis [33], predictive and prognostic stratification [34], phenotypic occurrence [35], and the effectiveness of professional interventions [36–39]. In other words, analytical workflows of data mining encompass the whole process of the disease course, from prevention, diagnosis, treatment, and finally to prognosis. Various types of clinical questions are resolvable through clinical data mining, but the most common are summarized in Table 1.

**Table 1:** Various clinical problems based on data mining

Type	Clinical data mining questions	Case	PMID
Disease prevention	What are the risk factors associated with the development of the disease?	Heart disease risk factors detection from electronic health records using advanced NLP and deep learning techniques	37138014
	Are there high-risk individuals who may benefit from preventive interventions or early screening?	A Cardiac Deep Learning Model (CDLM) to Predict and Identify the Risk Factor of Congenital Heart Disease	37443589
	How do lifestyle and environmental factors influence the likelihood of developing the disease?	The Contribution of Genetic Risk and Lifestyle Factors in the Development of Adult-Onset Inflammatory Bowel Disease: A Prospective Cohort Study	36695739
Disease diagnosis	What are the diagnostic markers or features that are most relevant for accurate disease identification?	Neutrophil-, Monocyte- and Platelet-to-Lymphocyte Ratios, and Absolute Lymphocyte Count for Diagnosis of Malignant Soft-tissue Tumors	37351995
	How can data-driven approaches be utilized to improve the accuracy of diagnostic tests or imaging techniques?	A semi-supervised multi-task learning framework for cancer classification with weak annotation in whole-slide images	36327654
	Does data mining have the ability to differentiate between different subtypes or stages of the disease?	Machine learning models based on immunological genes to predict the response to neoadjuvant therapy in breast cancer patients	35935976
Disease treatment	Which treatments or therapies are most effective for specific patient subgroups or disease stages?	Darolutamide Plus Androgen-deprivation Therapy and Docetaxel in Metastatic Hormone-Sensitive Prostate Cancer by Disease Volume and Risk Subgroups in the Phase III ARASENS Trial	36795843
	Can data mining be used to optimize treatment plans and personalize medicine based on individual patient characteristics?	Clinical Outcomes With and Without Plasma Exchange in the Treatment of Rapidly Progressive Interstitial Lung Disease Associated With Idiopathic Inflammatory Myopathy	36729874
	How do we predict treatment response and potential adverse reactions to specific medications?	Prognostic and predictive biomarkers for immunotherapy in advanced renal cell carcinoma	36414800
Disease prognosis	What are the key prognostic factors influencing disease outcomes and patient survival rates?	Construction and Validation of a UPR-Associated Gene Prognostic Model for Head and Neck Squamous Cell Carcinoma	35707371
	Can data mining assist in predicting disease progression and potential complications?	An inflammatory-related genes signature based model for prognosis prediction in breast cancer	37304237
	How can predictive analytics help to identify patients who are more likely to experience a recurrence or relapse of their disease?	Prognostic risk factor of major salivary gland carcinomas and survival prediction model based on random survival forests	36934429
Population health	How does data mining contribute to public health initiatives and disease surveillance efforts?	Perceived Impact of Digital Health Maturity on Patient Experience, Population Health, Health Care Costs, and Provider Experience: Mixed Methods Case Study	37463008
	What patterns and trends emerge when looking at the occurrence and spread of disease across different populations or geographic regions?	Analytical exploratory tool for healthcare professionals to monitor cancer patients' progress	36698423

### Raising and defining the clinical question

The clinical problem arising from clinical practice is the starting point and destination of clinical data mining. It is a common myth that the clinical problem for data mining is not natural, but rather artificial, due to a poor understanding of the clinical settings of the problem [23]. The deep reasons causing this problem lies in the fact that the complex processes of clinical decision-making are absent in the dataset for data mining [19]. By having access to shared clinical data, thousands of researchers can gain insight into a patient's treatment plan prescribed by the physician, yet without being privy to the rationale behind the physician's decisions and the factors they took into consideration. Consequently, it is essential to prioritize communication and collaboration with physicians on the clinical issue at the start of the research.

A true picture of the clinical problem directly determines the fate of clinical data mining results, whether the report is shelved after publication or takes root in clinical practice. And, the Cross-industry Standard Process for Data Mining, which is widely accepted, emphasizes understanding and grasping application scenarios above all else [40]. Thus, what kind of clinical problems can be solved and to what extent fundamentally determines the clinical significance of findings in clinical data mining. Therefore, only by effectively transforming clinical problems into data mining needs can we veraciously design data extraction of clinical characteristic variables, transparently establish a flowchart of statistical analysis, rationally select predictor parameters and significantly optimization target metrics, and iteratively implement predictive models, which requires that clinical thinking run through the entire data mining process.

**Fig. 1:**
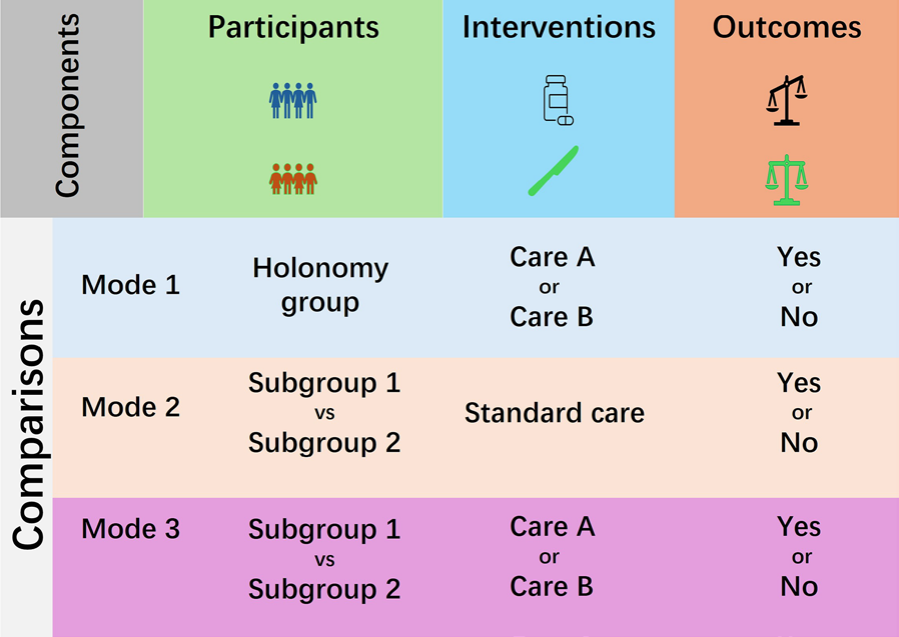
A schematic diagram for raising a clinical question based on PIOC. The clinical problem components include patients (P), interventions (I), outcomes (O) and comparisons (C). The comparative fundamental tenet shows the possibility of two or more interventions and outcomes

Raising and defining the clinical question is the core process of clinical data mining research [41, 42]. Reference to RCT design principles [43–45], a clinical question has three components: participants, interventions (absent in the diagnostic problem), and outcomes, plus one tenet of comparison, denoted by PIOC (Fig. 1). The size of the patients, the cost of the interventions, and the health damage of the disease all bind together to determine the potential of conducting the clinical problem. Special emphasis is placed on the fact that there are more than two standpoints to define the participants in a clinical problem, which a novice would not have the experience to differentiate. The reason behind this is that a diagnosis of a disease phenotype is made by a team of multiple clinical subspecialists, who are bound by certain disciplinary contexts consisting of inspection tools and angles, and by the applied pursuits of professionalism.

The outcome is defined by a set of measures using various subjective and objective tools and includes three subtypes: a measure of treatment effectiveness (rehabilitation or survival at three years), a measure of side effects (quantitative or qualitative), and a measure of patient trajectory by use of the professional scale following clinical guidelines. Beyond that together determining the optimal metric for prediction model training [46, 47], each subtype of outcome has its own clinical value; we urge investigators to first understand each one and then begin with the clinical practice need for data mining, even though this results in pooling data across institutions being challenging.

Above all else, the fundamental tenet of comparison is that both the intervention and the outcome variables have more than two possible values; that is, two or more treatments can be chosen for a patient, and then the outcomes of those patients could be either positive or negative. It is not appropriate to raise a research question involving participants who have only one available treatment or one treatment outcome. A schematic diagram for raising a clinical question is shown in Fig. 1.

### The multidimensional heterogeneity of treatment effectiveness

The most undeniable problem that we face in defining the clinical problem is having a thorough grasp of a high degree of dimensional heterogeneity in clinical practice reality. The heterogeneity facing data mining comes from two sources: the existing variation of patients in risk factor, genotype [48] and phenotype [49, 50], and the artificial variation of data capture by a measurement system [51–54] including devices, algorithms and definitions. Controlling the latter at a reasonable level is the precondition for identifying the former [55, 56] and is the surmountable challenge unsurprisingly hindering most model validations of data mining.

Then, closer than that, recognizing the former should be based on three dimensions: the temporal clinical practice following a continually updated clinical guideline [57], the spatial variation of the patient demographics [58–60], and the infinitely varied efficiencies of hospital operational systems [61, 62]. The first raises a requirement on the prospective validation of prediction models and endows the prediction model with a remarkable valid period [57, 63]. The second requires an off-site validation when the predictive model is going to be used outside of the original development place [64–67].

But the use of drugs or medical devices off-site is largely due to high costs, high risks, and long cycles for their development, and it does not seem to be necessary for the predictive model in a new era of big data. Consequently, in a certain sense, there was hardly a need to introduce a non-indigenous prediction model due to a very low overhead for developing the native one. Furthermore, the heterogeneity of hospital operational systems in terms of efficiency means that the same utilization of technological and material resources can lead to a variety of outputs, which determines the service quality of a hospital that can be expected to improve through prediction models.

These things above tremendously aggravate the problem of heterogeneity in treatment effectiveness, fostering great uncertainty for externally validating predictive models in clinical data mining. Table 2 summarizes the main heterogeneities in clinical practice for data mining in terms of participants, interventions, outcomes, and comparisons.

**Table 2:** The main heterogeneities in clinical practice for data mining

	Source of heterogeneity		Attributes
Participants	Demographic characteristics		Spatial heterogeneity; Time heterogeneity; Space–time heterogeneity
	Phenotype		
	Genotype		
	Behavioral characteristics and social factors		
Interventions	Proficiency in professional skills		Diversity of therapeutic regimen (monotonically improvement); Diversity of clinical practice guidelines
	Nursing quality		
	Medical quality		
	Accessibility of medical devices		
Outcomes	Type of the outcome: primary and secondary outcomes, side effects, disease progression		Subjectivity: doctor subjective report and patient self-report
	Definition of the outcome: including binary and continue with cut-off		Objectivity: diagnostic report (imaging, pathology, laboratory tests)
	Observation duration		Time effect: timeliness or lateness of outcome occurrence time
Comparisons	Case–control	PS Matching	Post-hot randomization
		PS Weighting (IPTW, SMRW)	Non-randomization
	Cohort studies	Instrumental variable	Randomization-like
	Randomization		Randomized controlled trial

*IPTW* inverse probability of treatment weighting, *SMRW* standardized morbidity ratio weighting

### The most efficient model in clinical practice

To manage heterogeneity effectively during model development, we suggest transforming a local clinical dataset into a prediction model that can be used by local doctors to manage their patients; namely the best models are those that are seamlessly deployed, markedly assisting with clinical decision-making, and improving the clinical paths in current practice. And one goal is to persuade you that internal validation is essential for confirming the predictive repeatability of analytic workflow using clinical data that originate from the same people as the training data. External validity, on the other hand, may not seem to be problematic, for being overly stringent and timid in clinical practice.

The assessment of verifying a predictive model in clinical data mining is a newer challenge. So far, there has been no scientific consensus about what constitutes the rule of externally verifying predictive model performance in clinical data mining, and about whether we need a unique set of standards for external validity. In our opinion, the precision of the prediction model based on data mining is more sensitive to the artificial variation of clinical data than the biomarker when conducting an external validity, while its cost is lower than that of the biomarker when conducting development. This review is not a discussion of these standards, nor does it end the discussion about them, but rather helps these standards evolve in a direction that is more adaptable to translation.

### The optimal transplantable workflow developing indigenized models

Clinical prediction models are typically the products of developing analytic workflows processing massive amounts of clinical data of various types based on computing power [13]. Internal and external validation do not necessarily guarantee the elimination of impacts from both natural and artificial variations, which inevitably impede the accuracy of clinical prediction models [24, 68]. In general, the external validation accuracy of clinical predictive models tends to decrease, as evidenced by the data in Table 3. But for now, we have identified that the internal validation of outstanding performance endorses the overall development process of a clinical predictive model [59, 69]. And the analytic workflows of developing predictive models, namely the process flow or workflow, are robust to these variations of clinical data.

**Table 3:** Accuracy summary of external validation for prediction model in clinical application scenario

Clinical scenarios	Model developer	Model feature	Training set		External verification set		PMID
			Optimal model accuracy (%)	Optimal model AUC	Optimal model accuracy (%)	Optimal model AUC	
Predicting pathological complete response after neoadjuvant chemoradiotherapy in LARC	Wei et al	Clinical-Imaging	99.8	1.0	86.3	0.872	36355199
	Defeudis et al	Imaging	83	0.90	68	0.61	35501512
	Bordron et al	Imaging	90	0.95	85.5	0.81	35205826
	Huang et al	Clinical	87	0.79	86	0.81	32724164
	Guo et al	Gene pairs	92.86	0.95	90.91	0.91	29402470

*LARC* locally advanced rectal cancer

Hence, we suggest that the current focus on transporting new models should shift to a focus on a transparently analytic workflow consisting of widely spread, feasibly conducted, and realistically assumed algorithms. In our view, some clinical problems might permit the development of general models, and some might merely allow general development workflows. There is no doubt that what allows a general model also allows a general workflow, but not vice versa.

This may be argued that their predictive model has already incorporated the knowledge and experience of professional doctors, as evidenced by the training clinical dataset. In response, we believe that by consulting these experts and revising our clinical practices, the quality of our clinical data will be significantly improved before training the predictive model.

## Aiming to identify clinically significant subgroups

Identifying clinically significant subgroups is a cornerstone of personalized medicine, enabling the tailoring of treatments to patient characteristics that influence therapeutic outcomes. In clinical practice, a subset of patients are more likely to gain benefit from the current treatment, outweighing the harm [70], whereas some are at a greater probability of the opposing situation [71]. Identifying a subgroup of patients with a unique eigenvalue or effect emerges continuously across a broad range of medical fields [72], often with the goal of delineating patient risk stratification and facilitating optimal decision-making for varied patients. In addition, the data of RCT studies failing to meet the primary endpoint can be reused to explore possible benefits in specific subgroups of participants [73]. And the subgroup poorly represented in RCTs, such as minorities of younger patients with comorbidities, is also found in adequate numbers to permit subgroup analyses in clinical data mining. Ultimately, identification of a clinically meaningful subgroup may lead to positive change in clinical practice [74], which is a sign of the success of the data mining.

### Discover or construct variables that define subgroups

There is no doubt that sharing clinical data offers a variety of opportunities to detect or discover a subgroup. By performing unplanned subgroup analyses, it is possible to uncover new hypotheses from clinical data mining. More critically, it will unmask that patients with severe comorbidities or vulnerabilities who have been excluded from RCTs have received different therapeutic benefits post-launch [75].

Leveraging a new variable to define clinical subgroups of patients is the core of developing a prediction model in clinical data mining. There are two ways in which one can use a variable to define a subgroup. The first is to directly use key baseline characteristics, including demographic variables such as age [76] and gender [77, 78], as well as important clinical phenotypes [79], such as disease severity [71] and comorbidities [80], to define the subgroups in a separate or combined manner. By conducting subgroup analyses based on natural features, it is possible to uncover the heterogeneity of the intervention effects among target patients, thus enabling the selection of those who would most likely benefit from the intervention. As an alternative, one can use a aposteriori variable to divide into subgroups, to identify subgroups with beneficial characteristics [81], such as improved therapeutic responses [34] or fewer treatment-related complications [82]. Researchers are increasingly reporting these created variables, such as polygenic scores [83, 84], on a continuous scale, enabling them to investigate how the effectiveness of treatment changes as the value of the novel variable increases [85–87]. Despite the potential of multivariable continuous models to reveal complex interactions, investigators should ultimately rely on binary subgroups that require a reasonable cut-off for a simpler explanation.

### Cut-offs of variables define subgroups

Keeping the same definition of patient subgroups allows for comparison of results between analogous subgroups in different research reports of clinical data mining. Utilizing continuous variables to define subgroups is a common practice, and it is recommended to use pre-existing or published cut-offs, as in reference [88]. As one embarks on the exploration, often there are no predetermined cutoffs for use in clinical data mining, as in reference [89].

The most classic example is that a new continuous predictive score of a clinical multivariable prediction model will be used to categorize patients into low and high risk of a benefited or adverse outcome [90–93]. Thus, the optimal cutoff point should be selected to maximize the disparity in outcomes or intervention benefits between the two subgroups. There are a couple of ways to specify the cutoff, and the most common, albeit inadvisable, one is that cutoff points are often identified groundlessly by simple percentiles, such as dichotomization using the median [94, 95]. By contrast, the better solution is to use the Subpopulation Treatment Effect Pattern Plot (STEPP) to identify the cut-off [96]. STEPP graphically explores the linear or nonlinear patterns of intervention effect across overlapping intervals of the definition variable of subgroups [96], in which the cutoff distinguishing the subgroups with different benefit patterns was determined.

### Exploratory and confirmatory subgroups

Clinical data mining enables two types of subgroup analyses: a confirmatory analysis that relies on a hypothesis (hypothesis-driven), and an exploratory analysis to build a hypothesis (data-driven). In confirmatory cases, the subgroups must be clearly predetermined on the solid evidence of hypotheses, and the endpoints must be established regarding the subgroup-specific treatment effects [97–100]. Moreover, a strategy limiting the type I error rate and ensuring adequate power for testing the subgroup treatment effects must be established before the research [99].

Exploratory subgroup analyses are conducted either post hoc [101] or prespecified at the design stage [102], although the latter usually lacks the strength to formally assess intervention effects [103]. When planning for prespecified exploratory subgroups analyses, one should consider the definition of the subgroup, the endpoints, and the method carrying out the subgroup analyses. The difference between confirmatory and exploratory subgroup analyses has been summarized in reference [104]. Recognizing the difference between them, we caution that both are equally important and work together [105] to form a complete entity.

### Matters needing attention in subgroup analyses

Subgroup analysis, however, increases the possibility of introducing bias and making interpretation more difficult on the variables that define subgroups. Consequently, it is vital to distinguish between the inexplicable subgroup analyses and those that are conducted appropriately. Certainly, it is improbable that a subgroup analysis will satisfy all or none of the current regulations for RCTs [106–108].

There is not yet a consensus on the value and significance given to each of these regulations in observational studies. Despite this, there is a high rate of methodological inadequacies in subgroup analyses [109, 110], especially in the illogical absence of statistical interaction tests [99] and arbitrary cut points for dividing subgroups [111]. In terms of testing the interaction, the multiplicative and additive interactions could have completely different impacts and clinical interpretations [112] so it is essential to understand how to properly conduct and interpret them.

We caution, however, that the statistical methods applied in current studies have not been adhered to as recommended. Firstly, when conducting subgroup analysis, the sample size should be adequate to robustly demonstrate the hypothesized subgroup effects. Additionally, a data-driven subgroup analysis should be accompanied by a hypothesis-driven subgroup analysis. Furthermore, the definition of subgroups should be based on the pathophysiology of the disease, its mechanisms, and high-quality internal and external data. Finally, and most importantly, caution should be taken when interpreting the findings of a subgroup analysis. Please refer to the literatures [72, 113–117] for more detailed methodological points.

## Evaluating the consequences of controlling confounders on the potential for bias in research findings

Clinical data mining complements RCTs by leveraging historical data to identify patient groups that may respond differently to existing treatments, thus enriching the evidence base for personalized care. Data mining of EHRs has been demonstrated to be able to replicate the findings of clinical trials [118–120], and to be more realistically assessed than in RCTs given their size and multifariousness of patients. However, it is unlikely to substitute but rather to be complementary to RCTs [121], for the justification that data mining is not as good at controlling imbalance confounders of unmeasured or crudely measured variables as RCTs.

### Swollen risk of confounding factors in clinical data mining

For clinical data mining studies, two analytical frameworks are available: a historical cohort study and a case–control study, both of which are observational in nature. Historical cohort studies require that the patient’s data be organized and presented over time, often referred to as an ad hoc database and a result of clinical data governance dealing with fragmented data storage [121, 122]. In contrast, case–control studies do not necessarily require any systematic data governance and are thus far more commonly used than historical cohort studies in clinical data mining.

Regardless of the analysis frameworks used in clinical data mining, researchers cannot randomize patients to receive treatment [123, 124]; rather, the patients have tendentiously received the treatments in past clinical practice [125]. Unfortunately, in most cases, this specific information on treatment selection is also missed or unrecorded in EHRs for clinical data mining. For instance, patients with a severe phenotype usually receive intensive treatment, yet this often leads to unfavorable results in practice, creating a misconception that intensive treatment yields poorer outcomes. This has led to the most common bias problem, namely, the treatment uptake mechanism introducing indication bias, which is a form of selection bias and occurs when selecting participants based on the presence of certain factors.

Moreover, compared to RCTs, data quality control is particularly intricate, and its impact is difficult to assess, inevitably resulting in the generation of bias, particularly in gathering non-structured clinical data from EHRs [119, 126]. Consequently, it is almost impossible to prevent confounding, or a threat to internal validity, without taking deliberate steps, including data collection and processing.

### Commonly used methods of propensity score

Comparison is essential in clinical research [127, 128], and the fundamental feature of various research designs is to identify the most comparable control group to the observed group [120, 129]. It is well known that confounders, which cause selection bias, are associated with both the intervention variable and the clinical outcome. Naturally, there are two approaches to reducing the effects of confounders: the Propensity Score (PS) and Mendelian Randomization (MR).

The PS seeks to create screening conditions [130], namely PS, for the observed and control groups that allow for secondary selection and make the two groups similar in terms of known confounding variables. A patient’s PS is a continuously distributed probability value, ranging from 0 to 1, of receiving the experimental treatment given the pretreatment confounding variables [131]. Hence, it is necessary to have knowledge and measurement of confounders, in addition to participants having a chance of being assigned either the observed or the control intervention [132].

To attain unbiased treatment effects in clinical data mining, PSs can be used in four ways: PS matching, PS hierarchy, PS correction, and PS weighting [133]. Their distinguishing features are outlined in the Table 4. Therein, two special reminders are needed: (1) In subgroup analyses, one must use the PS within each prespecified subgroup for matching or weighting; (2) The PS approaches are incapable when a confounder or intervention is time-varying, as is often the case for chronic diseases.

**Table 4:** Overview of four different PS-based approaches

Aspect	PS matching	PS hierarchy	PS correction	PS weighting
Principle	Matching one or more control cases with a propensity score almost equal to the PS for each treatment case	Stratifying the sample based on rank-ordered PSs and performing comparisons between groups within each stratum	Incorporating PS values as a covariate in regression analysis models	Utilizing the PS to develop weights and applying all outcomes of interest
Ability to control confounding bias	Superior to the PS hierarchy and PS correction	Weaker than other methods in particular to survival analysis	Weaker than PS matching and PS weight	Superior to PS hierarchy and PS correction
Data utilization	Removing data that does not match the study objectives	Retaining data from all study objectives	Retaining data from all study objectives	Retaining data from all study objectives
Causal effect estimation	Matching can estimate only the ATT	Hierarchy can estimate only marginal effect but neither the ATT nor the ATE	Correction can estimate only marginal effect but neither the ATT nor the ATE	Weighting can estimate either effect (ATT or ATE) according to the way weights are defined
Advantages	Addressing the confounding from multiple variables to guarantee equalization between individuals; The strength of the argument is strong and mirrors a closer randomized experiment	Achieving equilibrium of intergroup covariates within each stratum	Model-based analysis with a straightforward application	The strength of the argument is strong and mirrors a closer randomized experiment
Disadvantages	As only areas of the domain that are mutually supported by PS values can be matched, the sample size is reduced	Inadequate covariate equalization, particularly for the uppermost and lowest tiers	A model-dependent approach that can sometimes be challenging to meet the assumptions of the model	The sample in the study is only theoretical; Excessive weight will have an impact on the effect estimates

*PS* propensity scores, *ATT* average effect of the treatment on the treated, *ATE* average treatment effect

The PS method does not address the confounders directly; yet it is able to produce a randomization-like effect by re-recruiting participants to even out the two groups, thus diminishing or balancing the effect of the confounders on the results. Hence, it is also referred to as post-hoc randomization [134].

### Promising Mendelian randomization

Despite their efforts, PS methods are unable to address unmeasured confounders in clinical data mining. In clinical practice setting, if two patients with the same measured features receive different treatments, there may be valid but undocumented contributors [135]. Such items as extramural labs, clinical features, lifestyles, and cognitive and physical functioning that are obtained outside the hospital and affect both treatment decisions and outcomes are often not documented in HER. This presents a great opportunity to apply MR to clinical data mining.

MR is a poster-child example of the Instrumental Variable (IV) method [136], which can be used to determine the causal-effect estimates, even when unmeasured confounding is present [137]. The basic idea of MR is to find an instrumental variable that acts as a natural randomizer to mimic the obligation of randomizing the allocation of interventions [138]. For IVs to be valid, they must be able to affect treatment assignment, be independent of any measured or unmeasured confounders, and not have a direct effect on the interested outcomes [139], referred to as relevance, restriction, and independence.

In the opinion of experts, inherited genetic variants from parents can certainly be used as an excellent IV in research, as they are allocated randomly, remain unmodified, and are not influenced, perfectly suited to the rigors of these laws at conception [140]. MR can pinpoint the variant sites from thousands of options that fulfill the three criteria, thus making them a flawless IV. Moreover, utilizing multiple IVs which all point to the same conclusion would strengthen the persuasiveness of the evidence.

MR analysis consists of two steps: examining the three core assumptions and evaluating the causal effect between variables and outcomes, as in conferences [141, 142]. This process, where the two steps are carried out in the same sample, is known as one-sample MR [143], and when done in two distinct samples from the same population, it is referred to as two-sample MR [144]. MR is becoming increasingly popular among clinical data miners due to its time- and cost-effectiveness, largely attributed to the availability of chances to screen an abundance of published genetic associations [145]. Thus, genic IVs can be identified by searching through databases or reports that assess the relationship between genetic factors and the observed variable in question. Previous genome-wide association studies (GWASs) are especially useful in this regard [146, 147], as they are hypothesis-free scans that depict the correlation between millions of SNPs and the observed variables and clinical outcomes.

Yet, Mendelian randomization studies can be distorted by sample selection and misclassification if the observed variables are not universally measured with the same definition in all participants [148, 149]. Moreover, when utilizing MR, two aspects should be mulled over: (1) Population stratification, which involves the presence of subpopulations that are more likely to possess the genetic variant; (2) and the potential of the genetic variant used as an IV and its genetically linked genes to initially affect the outcome through a route other than the variable being observed [150–153]. Even so, it is important to bear in mind that the restricted access to dependable IVs and the minimal sample size may cause substantial finite sample bias and standard errors.

### What is indispensable may not have desired consequences

Clinical data mining studies are advantageous in terms of efficient design schemes, yet they can be biased if the control group fails to reflect the distribution of contributors in the population from which the participants were taken. Locating this population is usually difficult, and controls can be selected to a certain extent for convenience. We must be cognizant that no single control strategy is optimal for all clinical questions, and all of them have certain drawbacks.

In comparison to PS strategies, IV tactics may be more intricate and less explicable, yet can be more dependable in scenarios of unmeasured confounders. Meanwhile, unless there is evidence that the controlling confounders approach will be significantly impacted by unobserved confounding, PS approaches should be preferred in clinical data mining. More importantly, in this paper, we seek to raise awareness that both approaches are equally necessary for obtaining precise intervention effects that vary across subgroups, as observational data is being increasingly used. Researchers must understand the basic suppositions of these approaches and the situations in which these approaches are most suitable, as unavoidable examples leading to distorted estimates in the literature still exist [154–156].

## Vigilance and mitigation of increased false positives due to multiple hypothesis

**Fig. 2:**
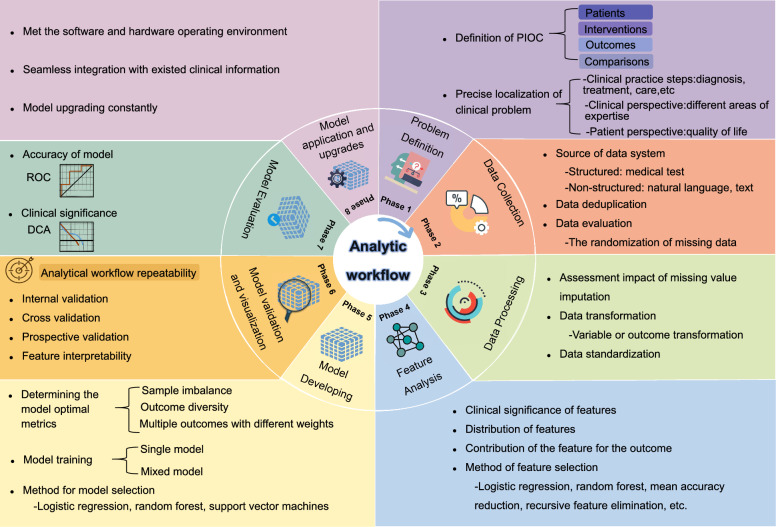
Clinical data mining studies analyze workflows. The analytic workflows for developing a model consist of eight steps, including phase 1: problem definition (show in purple); phase 2: data collection (show in orange); phase 3: data processing (show in light green); phase 4: feature analysis (show in blue); phase 5: model developing (show in light yellow); phase 6: model validation and visualization (show in dark yellow); phase 7:model evaluation (show in dark green); phase 8: model application and upgrades (show in pink)

The concept of false positive findings has a long history in statistics, and especially in data mining, is far from trivial [157, 158]; indeed, they can cause unsafe prediction failures. Until now, this essential issue, which greatly determines the success of clinical data mining, has not been given sufficient attention. Clinical data mining studies strongly involve numerous analytic steps (Fig. 2), and at each step, hypotheses must be thoroughly evaluated, thereby leading to a heightened risk of false positives due to multiple hypothesis tests [159, 160].

### Intentional or unintentional multi-testing

From a data scientist's point of view, the near exhaustiveness of data analyses is advantageous; however, it also means that coincidental random fluctuations can be misinterpreted as significant changes in the clinical practice context, resulting in erroneous positive results and potentially deceptive conclusions. In clinical data mining research, concerns about excessively bloated rates of false-positive findings have led to a serious lack of confidence in prospectively validating results, incurring costs in money and time; this is the main reason why translational application is few and far between. In the following, we enumerate the more prevalent types.

Clinical data mining involves multiple comparisons of clinical outcomes [161], such as assessing if a selected clinical outcome differs between more than two intervention groups [162], or which of these outcomes vary between two intervention groups [163], especially in data from basket and umbrella trials [164]. Simultaneously, clinical data mining screens frequently clinically relevant variables or IVs, with multiple judgments being made to determine whether thousands of characteristics, such as genomic nucleotide site polymorphisms [165], transcriptomes [166], proteomes [167], metabolomes [168], and microbiomes [169], are linked to a certain observed variable or endpoint [170]. As the definitions of subgroups and endpoints become more intricate in clinical data mining, this issue is becoming more complex as well.

### Extremely inflated false positives in data mining

A false-positive result is a risk with any statistical test, as it is caused by chance rather than any difference between the comparison groups [171]. This means that if the original hypothesis is rejected, a Type I error has been made. Unexpectedly, as the number of tests increases, the likelihood of a false-positive result also rises surprisingly [172]. To ensure that the total number of Type I errors remains below a predetermined level, appropriate methods must be employed.

Benjamini and Hochberg were the first to introduce the concept of False Discovery Rate (FDR) [173], which is the proportion of false positive test results [174]. They also proposed a corresponding control method, known as the BH method. Compared to Type I error correction, FDR can be adjusted according to the needs of data mining and used as a criterion for variable selection or feature extraction.

We strongly recommend taking note that FDR is calculated based on the P-value under certain conditions that the assumptions are independent of each other [171]. The extensive existence of correlations between variables or outcomes is always inconsistent with the assumptions mentioned above, thus FDR does not guarantee a finding in fact, but rather provides a conservative approach in statistics. Before coming to any sweeping conclusions, it is imperative to understand the statistical caveats and limitations of the approaches [175, 176].

## Focuses and outlooks

Over the past decade, technological advances in data have greatly enhanced our ability to stockpile and revisit complex processes of clinical diagnosis and treatment on a large scale and to be in ascendance. The sheer volume of clinical data has necessitated the utilization of data mining methods that are being specifically upgraded in tandem. Clinical data mining is largely aimed at clinical scenarios of actual practice, in which unrestricted patients range from whole genotypes to whole phenotypes and are tendentiously given various treatments in a non-randomized manner. And with data collection in the glare of scientific organization, RCT presents a new source of clinical data that is measured on-demand. Tying the two together, data mining has the potential to produce an enhanced version of the findings and is therefore expected to yield valid evidence for medical decision-making.

The limited translational application prevents any improvements from being seen in clinical practice. Various standards and guidelines have been suggested by academics, yet their impact has not been perceptible. The standards, to some degree, are too rigorous to be met, and there is no agreement among them as to whether the same standards of pharmaceuticals or medical devices should be applied. Perhaps it may take some time to observe the positive impacts of the existing regulations. But we all know deep down that the omnipresent heterogeneity of curative effects and prognoses is not a temporary bet, but a major impediment to their use in long-span populations, making it difficult for many predictive models of clinical data mining to match our field observations.

The time is right for a new doctrine. More concretely, to satisfy the needs of translational applications, we propose that adjustments must be made to the principles of clinical data mining. Most importantly, the present conception, namely PIOCs, maximizes the significance of clinical issues that are defined in clinical data mining. Subsequently, systematic analysis of the effect of heterogeneity on each of the PIOCs interprets as much as possible the failures of the translational application of predictive models in clinical data mining. Recognizing these, this review has devised a strategy for contributing to the speed-increasing gearbox of translational applications by prioritizing the execution of development analytic workflows from clinical data mining in the future. In other words, clinical data mining research should focus on recognizing and assessing data inconsistencies and confirming the analytic procedure and its executable files employed to develop the predictive model, instead of simply popularizing the developed model. Rather than simply providing predictive models, the sharing of analytic processes for creating them should be more widespread in the same medical field among hospitals. In short, by utilizing external but credible analytical workflows, clinical data mining employs local data to train an indigenized model to play an auxiliary role in a clinical specialty during a period in local hospitals, upgrading the predictive model when clinical practices isomerize significantly.

Of these, identifying the subgroup of patients with markedly different intervention results or risk of side effects can be achieved by using the natural variables or constructing novel variables to leverage subgroup analysis. In this process, one must address these creeping cracks of potential bias due to the non-randomization of the intervention and patient characteristics, and we propose a set of heuristics to help select the most suitable method that compromises those assumptions to the least extent. And if there is a pitfall to address, it would be the false positives, reducing which demand us gathering information on the background of the FDR and its resolution in clinical data mining.

It is the responsibility of clinical decision makers to develop personalized prediction models that are transparent, clinically effective, and beneficial for the patients they are caring for. Most importantly, an analytic workflow developing a tailored model for the data-mined evidence is practicable for decision makers now. Automatically, the data-mined evidence will be employed by clinicians to make distinct prescribing decisions without any doubt. As the translation of single-hospital discovery to single-hospital application is being increased, an increase in the accessibility of clinical data and analytical codes [157, 177] combined with a mitigation of conceptual and metric shifts for PIOCs [178, 179], guarantees that precision medicine will reach its full potential. Moreover, precise models hold significant potential in the current biomedical field. In contrast to the majority of application universality models, our CSCF framework emphasizes diversity and personalization of models. We believe that personalization and accuracy of the model should not be compromised for the sake of generalization. The CSCF framework ensures that the model is truly customized to the specific needs of patient populations or geographic regions by considering factors such as the clinical context, subgroups, confounders, and false positives, leading to a deeper understanding of disease mechanisms. This targeted approach not only improves the predictive accuracy of the models, but also ensures their usefulness and operability in clinical settings. By promoting diversity and personalization of models, we can avoid the trap of overgeneralization, thereby preventing a loss of correlation between model accuracy and specific patient populations. We are certain that clinical professionals will be able to increase the curative quality they offer due to the implementation of clinical data mining, and this will remain true in the days to come. Clinical data mining will not substitute for clinical professionals, but rather will facilitate them to carry out their duties more effectively and give them more time to collaborate with data scientists, exchange ideas with their peers, and engage with patients.

## Data Availability

This article does not involve any research data or code.
